# The Role of Diet in Functional Dyspepsia Management

**DOI:** 10.3389/fpsyt.2020.00023

**Published:** 2020-02-05

**Authors:** Henri Duboc, Sofya Latrache, Nicoleta Nebunu, Benoit Coffin

**Affiliations:** ^1^Université de Paris, Paris, France; ^2^AP-HP, Gastroenterology Unit, Hopital Louis Mourier, Colombes, France; ^3^INSERM UMR 1149, Université de Paris, Paris, France

**Keywords:** functional dyspepsia, diet, eating behaviour, fat, gluten, FODMAPs, alcohol

## Abstract

Functional dyspepsia is a common functional gastrointestinal disease that is characterized by postprandial fullness, early satiation, epigastric pain, and/or epigastric burning. Eating a meal is a key factor in the occurrence of symptoms during functional dyspepsia, and patients frequently request dietary advice that could relieve these symptoms. Eating behaviors, irregular meal patterns, and moderate-to-fast eating rates are significantly associated with functional dyspepsia. The role of diet is complex; fat ingestion increases the occurrence of symptoms in dyspeptic patients, which might be affected by cognitive factors and palatability. Data concerning the role of carbohydrates are conflicting. Wheat may induce symptoms in patients with nonceliac gluten/wheat sensitivity, and gluten-free diets might be beneficial. Data concerning the role of FODMAPs (Fructo, Oligo, Di-, Monosaccharides, And Polyols) in functional dyspepsia are lacking; however, as there is a frequent overlap between functional dyspepsia and irritable bowel syndrome, a diet that is low in FODMAPs might be useful in relieving some symptoms. Data concerning alcohol are also conflicting. Adherence to a Mediterranean diet seems to be associated with a decrease in dyspepsia symptoms. Finally, data concerning diet modifications are conflicting, and the impact of diet modifications on symptom intensity or frequency has never been reported in randomized prospective studies. Common sense dietary recommendations, such as eating slowly and regularly, as well as decreasing the fat content of meals, can be provided in daily clinical practice.

## Introduction

Functional dyspepsia is a common functional disease that affects up to 20% of the population, and it is believed to originate from the gastro-duodenal region (1). According to the Rome IV criteria, functional dyspepsia is defined by one or more of the following symptoms: bothersome postprandial fullness, bothersome early satiation, bothersome epigastric pain, and/or bothersome epigastric burning, with no evidence of structural diseases, including the use of an upper endoscopy (if necessary), according to age, past history, or presence of alarm symptoms in the patient (2). Symptoms must be present for at least 3 days a week during the last 3 months and must be chronic, with an onset of at least 6 months before the diagnosis. Two subgroups of dyspepsia have been identified. Postprandial distress syndrome is defined by bothersome postprandial fullness, such as fullness that is severe enough to have an impact on typical activities, and/or bothersome early satiation, such as satiation that is severe enough to prevent the completion of a regular size meal. Epigastric pain syndrome is defined by bothersome epigastric pain and/or epigastric burning, which are both severe enough to have an impact on usual activities (2). In most patients, there is a temporal relationship between meal ingestion and the occurrence of symptoms during postprandial distress syndrome and during epigastric pain syndrome, but also symptoms are not necessarily associated with a meal, as pain can be induced or relieved by the ingestion of a meal or may occur during fasting (2).

## Functional Dyspepsia and Meal Ingestion

Meal ingestion is clearly a triggering factor of the symptoms in dyspeptic patients. In a cohort of 218 patients with functional dyspepsia, Bisschops et al. demonstrated that the intensity of dyspeptic symptoms occurred rapidly (within 15 min) after ingestion of a test meal and remained elevated until the end of the measurement period (4 h) (3). The time course of the development of the individual symptoms varied, with early peaks for fullness and bloating, intermediate peaks for nausea and belching, and late peaks for pain and burning (3). Meal-induced aggravation of symptoms was reported by 79% of patients and was not associated with a decrease in the gastric-emptying rate, as only 20% of the patients had a delayed gastric emptying. These results suggest that factors other than gastric motility may explain the development of symptoms. Hypersensitivity to gastric distension has been demonstrated as one of the key pathophysiological factors in patients with functional dyspepsia during fasting and during the postprandial period (4, 5). In this latter, gastric distension was demonstrated to induce more intense symptoms in patients than in control individuals and to reproduce spontaneous symptoms. The highest symptom severity scores were obtained for postprandial fullness and bloating, whereas the lowest score was obtained for epigastric burning (5). Gastric distension was also associated with impaired gastric accommodation to meals (5). Finally, Di Stefano et al. demonstrated that, in comparison to healthy controls, gastric postprandial hypersensitivity and relationship between symptoms intensity and postprandial discomfort threshold were significant only in patients with postprandial distress syndrome and not in patients with epigastric pain syndrome (6). As eating a meal is a key factor in the occurrence of symptoms during functional dyspepsia, patients frequently request dietary advice that could relieve these symptoms.

## Eating Behavior and Functional Dyspepsia

In normal conditions, meal ingestion induces fundic accommodation with subsequently slow contractions, antral contractions, and finally, gastric emptying. As it has been previously reported, gastric fundic accommodation, as measured by an electronic barostat, is significantly impaired in patients with functional dyspepsia (5). A rapid drinking test, which is a noninvasive test, has been proposed as a diagnostic method to evoke the symptoms of functional dyspepsia, and it has been shown that the occurrence of dyspeptic symptoms is related to impaired gastric accommodation (7). In the daily lives of patients, abnormal eating behaviors, such as rapid or large volume meal ingestion (conditions that are reproduced during the rapid drinking test), may overload the gastric accommodation process, thus generating symptoms. In small cohorts of patients, the results have been conflicting, with some authors observing a relationship with dietary patterns, such as eating quickly, and the occurrence of symptoms (8, 9), whereas other authors have not observed this relationship (10). Recently, in a large cohort of 4,763 Iranian adults, and through the use of the technique known as latent class analysis (which is a person-centered approach that provides a unique opportunity to classify individuals according to behavioral subclasses), Keshteli et al. (11) found a prevalence of dyspepsia of 15.2%. Furthermore, these authors could identify that irregular meal patterns [odds ratio (OR): 1.42; 95% CI: 1.12–1.78] and moderate-to-fast eating rates (OR: 1.42; 95% CI: 1.15–1.75) were significantly associated with chronic uninvestigated dyspepsia. Meal-to-sleep intervals and intrameal fluid intake, which were the other two domains that were investigated, were not associated with dyspepsia (11). This study has some limitations as the evaluation of patients was made only by questionnaire, the use of translated modified Rome III questionnaire, and the lack of systematic endoscopic evaluation, but it confirms the observations that were made during the provocative tests. The physiology of food intake is complex; besides gastric distension induced by volume of meal ingestion, meal temperature may also modify gastric perception as cold temperature may induce smooth muscle contraction (12). In a small study of patient with epigastric pain syndrome, Wang et al. (13) demonstrated that gastric perfusion with an 8°C liquid induced significant gastric contraction and decreased gastric sensory threshold in comparison to a 37°C liquid infusion. Even if no interventional studies have been reported so far, we can still recommend that patients eat slowly (which is probably more important in the subgroup of patients with delayed gastric emptying) and regularly and probably avoid cold liquid ingestion.

## Nutrients and Functional Dyspepsia

### Fat

Dietary fat is ingested in a number of different forms, depending on the type of food that is eaten (i.e., extracellular vs. intracellular fat), the meal temperature (solid fat vs. oil), and the proportion of fat ingested with other macronutrients. A high-fat diet is frequently associated with high carbohydrate content or high protein content. In normal conditions, a high-fat meal is associated with a decrease in the gastric emptying rate (14). A prospective cross-sectional study that was performed in delegates attending a conference on four consecutive days demonstrated that a low-fat, low-calorie dinner induced significantly fewer symptoms than a high-calorie dinner (15). During a mechanistic study with gastric distension, intraduodenal fat infusion, but not an infusion with carbohydrates, induced a greater intensity of symptoms in dyspeptic patients (16). In an experimental condition with a similar calorie intake, a high-fat meal induced more symptoms than a high-carbohydrate meal in dyspeptic patients, with symptoms including nausea, bloating, postprandial fullness, and epigastric pain (17). Carvalho et al. (18) reported no differences in the total caloric intake between 41 patients with functional dyspepsia and 30 healthy controls; however, these authors found a significant reduction in fat intake (28% vs. 34%). These results were not confirmed by other authors (19). Cognitive factors may also contribute to the occurrence of symptoms by fat ingestion in patients with functional dyspepsia. In a randomized study, Feinle-Bisset et al. demonstrated that high-fat foods elicited more symptoms than low-fat foods; however, low-fat foods elicited similar symptoms if the patients perceived these foods to be high-fat foods even if they were not (20). Finally, meal palatability may also interfere with the occurrence of symptoms (21). No interventional study testing the long-term effects of a low-fat diet on symptoms has been reported so far. It is likely that dyspeptic patients, especially patients with severe symptoms, have determined for themselves that lipids can increase their typical symptoms. Therefore, these patients will spontaneously decrease their degree of fat ingestion; if they do not adjust their diet, then a low-fat diet could be recommended.

### Proteins and Gluten

No study has reported a relationship between symptoms and the intake of proteins. Functional symptoms, irritable bowel syndrome (IBS), and dyspepsia are frequent occurrences in patients with celiac disease, with an OR of 4.48 for biopsy-proven celiac disease in patients fulfilling the Rome criteria for IBS (95% CI: 2.33–8.60) (22). However, the risk of celiac disease in patients with functional dyspepsia does not seem to be increased (23). Conversely, during an observational study in a cohort of 85 patients with diagnosed celiac disease, 27% of the patients fulfilled the criteria of functional dyspepsia upon inclusion, and only 8% of the patients remained dyspeptic after consuming a gluten-free diet for 1 year (24). More recently, the concept of nonceliac gluten/wheat sensitivity has emerged in the literature (25). Nonceliac gluten/wheat sensitivity is a syndrome that is characterized by intestinal and extra-intestinal symptoms related to the ingestion of gluten-containing food, and this syndrome occurs in subjects who are not affected by either celiac disease or wheat allergies (26). Dyspeptic symptoms are frequent in patients with nonceliac gluten/wheat sensitivity, with approximately 50% of patients having reported nausea or epigastric pain in an Italian cohort (27) and 31.3% in an Australian cohort (28). Patients having dyspepsia reported more frequently symptoms of postprandial distress syndrome (26%) than epigastric pain syndrome (17%), and differences for both subtypes were significant in comparison to the control group (postprandial distress syndrome: OR 2.98, CI 95%: 2.34–3.79; epigastric pain syndrome: OR 3.17, CI 95%: 1.31–3.50) (28). In these patients, Elli et al. demonstrated that, during a double-blind crossover study, gluten-free diets induced a significant decrease in postprandial fullness, early satiety, and epigastric pain (29). However, this study has some limits as symptoms were evaluated by visual analogic scale and not specific questionnaire, and also in the whole group of patients and not only in those identified with isolated dyspeptic symptoms. However, there is increasing popularity in the general population that a gluten-free diet may be “healthier,” despite a lack of evidence to support this notion, as 8%–16% of the western population adheres to a gluten-free diet, which has resulted in a gluten-free food industry boom of an estimated $6 billion per year (25). The long-term nutritional impact of a gluten-free diet is still a matter of debate; adults adhering to a gluten-free diet did not consume enough nutrient-dense foods to meet all nutritional recommendations (30). Also, some patients who begin avoiding dietary gluten with the intention of improving their health and well-being may ultimately progress to develop pathologically obsessive behaviors regarding their diet (30). Results of well–conducted, randomized, controlled clinical trials testing the effects of gluten-free diet in dyspeptic patients are still lacking to clearly recommend such diet in patients. In daily clinical practice, if a diagnosis of celiac disease has been ruled out with a negative test of anti-transglutaminase antibodies and if a dyspeptic patient asks for the efficacy of gluten-free diet, then a short (4–8 weeks) gluten-free diet test period could be suggested, but this diet should be continued only if the symptoms decrease significantly according to patient's perception with a good clinical evaluation.

### Carbohydrates

Only three studies have examined the relationships between carbohydrates and dyspeptic symptoms, with conflicting results. One study reported a lower carbohydrate intake being associated with the occurrence of symptoms (31), which was possibly related to the lower energy intake that was reported in this study. Another study reported a daily intake of carbohydrates in dyspeptic patients in comparison to controls (230 vs. 199 g/day), but the difference was not significant (18), and one study reported no relationship between symptoms and a high-carbohydrate meal (17). Thus, no specific recommendation can be made.

## Fodmaps and Functional Dyspepsia

FODMAPs (Fructo, Oligo, Di-, Monosaccharides And Polyols) are poorly absorbable and highly fermentable substances that may induce bloating and gas sensations, which are common symptoms in dyspepsia, even if they do not specifically appear in the Rome IV diagnostic criteria. By using specific dietary questionnaires, it has been shown in several studies that dyspepsia is associated with grain/pasta/wheat products, soft drinks/carbonated drinks, fruit/fruit juice/watermelon, milk, and takeout/processed foods (e.g., pizza/fried foods) (32). Most of these foods contain a large proportion of FODMAPs. Several studies have clearly demonstrated that a low-FODMAPs diet significantly decreases symptoms in IBS patients (33). The overlap between IBS and dyspepsia is frequent and, in daily clinical settings, the overlap of both of these syndromes has occurred in 64% in patient questionnaires vs. 23% in routine clinical documentations (34). Thus, this overlap appears to be a normal occurrence, rather than an exception. However, the intensity of dyspeptic symptoms has never been specifically reported during the clinical studies that reported a beneficial effect of a low-FODMAPs diet in IBS patients (33). Indeed, further randomized studies that are specifically performed with dyspeptic patients are needed to recommend a low-FODMAPs diet in dyspeptic patients as during IBS. However, it could be suggested in some dyspeptic patients with IBS symptoms and/or bloating, with a 4- to 8-week test period.

## Ultra-Processed Foods and Functional Dyspepsia

Ultra-processed foods (UPFs) are industrial formulations that are usually made from substances derived from foods and additives by using a multitude of sequential processes to create the final product (hence the term “ultra-processed”). UPFs are typically branded, convenient (durable and ready to consume), and hyperpalatable food products that tend to displace fresh or minimally processed foods, as well as freshly prepared dishes and meals. UPFs are characterized by a high density of saturated fatty acids, sugars, and sodium, as well as a low content of fiber. In a large cohort of 33,343 subjects from the web-based NutriNet-Santé cohort who responded to dietary and Rome III questionnaires, Schnabel et al. demonstrated that the proportion of UPFs in the diets was not associated with symptoms in subjects who had pure functional dyspepsia (OR: 1.066; 95% CI: 0.97–1.16), whereas it was significantly associated with symptoms in patients who had IBS (OR: 1.09; 95% CI: 1.04–1.14) and in patients with associations of both IBS and functional dyspepsia (OR: 1.14; 95% CI: 1.05–1.24) (35). No interventional studies have been reported; however, we can recommend that not only patients but also the whole population should decrease the intake of UPFs, which also include additives, contact material, or neo-formed contaminants frequently not or poorly detailed in their formula (35), and should increase the intake of fresh and tasty foods.

## Alcohol and Functional Dyspepsia

Alcohol interferes with normal gastric physiology. Alcohol can increase gastric acid secretion, with low doses accelerating gastric emptying and high doses delaying gastric emptying (36). When considering the effects of alcohol on dyspepsia, the results are also conflicting. Some studies have not demonstrated any relationship between the occurrence of new dyspeptic symptoms and the severity of dyspepsia, postprandial distress syndrome, or epigastric pain syndrome (regardless of the subgroups) (37, 38). On the other hand, a large cohort study demonstrated that in 4,390 subjects, there was a relationship between the consumption of greater than seven alcoholic drinks a week and dyspeptic symptoms (OR: 2.3; CI 95%: 1.1–5.0) (39). Thus, it is difficult to determine if alcohol induces or not dyspeptic symptoms. As chronic alcohol consumption is not healthy, we can recommend decreasing the level of alcohol consumption in dyspeptic patients, as in all other conditions, with a maximum intake of 10 units a week in both men and women according to the WHO recommendations (40).

## Spicy Foods

Capsaicin is the active component of spicy foods (chili, red pepper …). It has a dual effect with first activation of C-afferent fibers, sensitization that increases symptoms, followed by desensitization that decreases symptoms. Capsaicin test, that is, ingestion of 0.75 mg capsule of capsaicin, has been proposed as a simple diagnostic test to reproduce symptoms in the subgroup of dyspeptic patients with gastric chemosensitivity (41, 42). A randomized study with only 30 patients (15 in each group) demonstrated that oral supplements with 2.5 g red pepper powder during 5 weeks induced a significant decrease in dyspeptic symptoms overall and also epigastric pain or fullness; two patients of the red pepper group had to rapidly stop the study because of an increase of abdominal pain (43). There is no sufficient convincing clinical data to recommend the chronic ingestion of spicy foods to decrease symptoms in dyspeptic patients; however, we can strongly recommend avoiding occasional intake of spicy foods, which probably could increase symptoms.

## Coffee and Functional Dyspepsia

Coffee consumption increases gastric acid secretion (44). Coffee consumption has also been found to induce dyspeptic symptoms (8, 18, 44), whereas in one study, no association was found (37). Thus, it is difficult to conclude on the effects of coffee consumption on dyspeptic symptoms, but patients have been observed to frequently and spontaneously decrease coffee consumption.

## Is There a Good Diet for Dyspeptic Patients?

The relationships between diet and dyspepsia complaints are complex and, except for fat, no clear recommendations based on randomized trials can be proposed to patients. Common sense advice can be provided to the patients, with recommendations including slow and regular eating, decreasing fat intake, possibly testing a gluten-free diet or a low-FODMAPs diet over short-term periods, avoiding coffee, and having a low alcohol intake (**Table 1**). Recommendations in observing a Mediterranean diet could also likely be performed. A Mediterranean diet is considered to be a complex set of eating habits adopted by people in countries bordering the Mediterranean Sea. This diet includes a high consumption of olive oil, fiber-rich foods, milk, or dairy products, in addition to a low consumption of meat or meat-based products. In the last few years, this dietary regimen has been proposed as a health-protective diet because populations who have adopted it exhibit a remarkable reduction in all-cause mortality, especially from cardiovascular diseases and cancer, and when compared to the United States or Northern European countries. Recently, *via* the use of a questionnaire that measured adherence to a Mediterranean diet, Zito et al. (45) demonstrated that in a population of 1,134 subjects, a lower adherence to a Mediterranean diet was significantly associated with the occurrence of dyspepsia (adherence score: 0.56 ± 0.24) and of IBS (adherence score: 0.57 ± 0.23), in comparison to controls (adherence score: 0.62 ± 0.21), mainly in the 17–24 and 25–34 age groups. With increasing age, patients have tended to adopt dietary regimens that are more similar to Mediterranean diets and, consequently, have fewer symptoms. From this observational study, it cannot be concluded if Mediterranean diet will decrease symptoms by itself or if it has a preventive effect on the occurrence of dyspeptic symptoms.

**Table 1:** Common sense dietary recommendations that could be provided to patients with functional dyspepsia.

Eat slowly and regularly.
Decrease fat intake.
Try to observe a diet that is more similar to a Mediterranean diet or increase the intake of fresh foods and decrease the intake of ultra-processed foods.
Decrease coffee and alcohol consumption.
A gluten-free diet and a low-FODMAPs diet could be tested over a short time period (4–8 weeks) and must be stopped if there is no efficacy.
Be careful in providing strong recommendations to obsessive patients, and avoid the recommendation of very restrictive diets.

No interventional studies have been reported thus far.

## Limits of Dietetic Recommendations in Patients With Functional Dyspepsia

In patients with functional dyspepsia, weight loss is considered an alarm sign that much leads to complementary examinations (2). However, in dyspeptic patients referred to tertiary referral centers, weight loss >5% is not exceptional as it can occur in around 40% of patients with epigastric pain syndrome as well as postprandial distress syndrome (46). In this study, weight loss was significantly higher in patients with early satiety and vomiting. It is highly probable that weight loss occurred because patients limit, consciously or unconsciously, their oral intake to decrease symptom intensity. In this condition, dietetic approach is limited, and specialized advice with dietician must be performed in order to try to regain weight.

## Conclusion

Food is clearly a triggering factor for dyspeptic symptoms in the majority of patients. However, the relationships between nutrients, except for fat, or other specific foods and the onset or intensity of dyspeptic symptoms have been poorly evaluated, and there is a lack of high-quality evidence to guide dietary therapies in functional dyspepsia. The effects of a gluten-free diet or a low-FODMAPs diet could be tested during interventional studies. Large cohort studies are also necessary to better identify the relationships between food and dyspepsia. As no clear recommendations are available, only common sense dietetic recommendations can be provided during daily clinical practice (**Table 1**). However, we must be very cautious, as some obsessive patients may observe very restrictive diets inducing nutritional deficiency.

## Author Contributions

HD and BC wrote the manuscript. SL and NN made the literature review.

## Conflict of Interest

The authors declare that the research was conducted in the absence of any commercial or financial relationships that could be construed as a potential conflict of interest.

The handling editor declared a past collaboration with one of the authors BC.
